# A review of methods to assess the economic impact of distributed medical education (DME) in Canada

**Published:** 2018-03-27

**Authors:** Kim Lemky, Pierre Gagne, Jill Konkin, Karl Stobbe, Gervan Fearon, Sylvia Blom, Geneviève Maltais Lapointe

**Affiliations:** 1 Brandon University, Manitoba, Canada; 2 Campus de l’Université de Montréal en Mauricie, Faculté de médecine, Université de Montréal, Québec, Canada; 3 Divison of Community Engagement, Edmonton Clinic Health Academy, University of Alberta, Alberta, Canada; 4 Niagara Regional Campus, Michael G. DeGroote School of Medicine, McMaster University, Ontario, Canada; 5 Brock University, Ontario, Canada; 6 Charles H. Dyson School of Applied Economics and Management, Cornell University, New York, United States; 7 Institutional Data & Analysis, Brandon University, Manitoba, Canada

## Abstract

**Background:**

Canadian distributed medical education (DME) increased substantially in the last decade, resulting in positive economic impacts to local communities. A reliable and simple method to estimate economic contributions is essential to provide managers with information on the extent of these impacts. This review paper fills a gap in the literature by answering the question: What are the most applicable quantitative methods to assess the economic impact of Canadian DME programs?

**Methods:**

The literature is reviewed to identify economic assessment methods. These are evaluated and compared based on the benefits, challenges, data needs, outputs and potential for use in the DME context.

**Results:**

We identified five economic impact methods used in similar contexts. Two of these methods have the potential for Canadian DME programs: the Canadian Input-Output (I-O) model and the Simplified American Council on Education (ACE) method.

**Conclusion:**

Choice of a method is contingent on the ability to measure the salient economic impacts, and provide an output that facilitates sustainable decision making. This paper thus fills a gap by identifying methods applicable to DME. These methods will assist stakeholders to calculate economic impacts, resulting in both the advancement and sustainability of these programs over short-and long-term time frames.

## Introduction

Distributed medical education (DME) is a term that describes medical education conducted outside of traditional academic health centres and urban medical school campuses; it is a broad grouping that also includes regional medical campuses (RMC) or decentralized medical education.^1,2^ Increasing the training opportunities for medical doctors outside of large urban centres reduces the disparity in medical services between rural and urban areas.^3-5^ Medical school graduates are more likely to remain in the region where they trained.^6^ Hence, the development of training opportunities in underserved areas mitigates the shortage of physicians.^6-8^ DME programs yield social and health benefits as well as economic benefits to the communities they serve.^8^

An economic impact is measured as the net changes in new economic activity associated with an industry, event or policy in an existing regional economy.^9^ DME brings new revenue into a region and it can keep revenues in a region that might otherwise be lost.^9^ Economic impacts are measured as the amount of money generated through direct, indirect, or induced impacts. Direct economic impact, is the initial spending or dollar value input into the economy by the university. Indirect spending occurs when the initial expenditures are spent again by the recipients (university employees) and businesses. Induced impacts occur when those businesses make their own purchases and hire employees who also spend salaries and wages throughout the local economy. This chain of direct, indirect, and induced spending continues with subsequent rounds of additional spending gradually diminished through savings, taxes and expenditures made outside the region or province.^10^ A community with health services typically makes it more attractive to businesses (health-related or otherwise) considering a relocation or expansion.^11^ There was a five-fold increase in student enrolment in DME programs (from 152 to 734 students) between 2005 to 2009,^1^ in Canada, and there is understandable stakeholder interest in estimating the economic impact of this growth.

The economic benefits of DME programs are both quantitative and qualitative. The quantitative benefits are often measured through economic impact studies while the qualitative are often considered through expectations or perceptions of the community,^12,13^ the impact of preceptors’ time and productivity,^14-17^ and student employment outcomes,^18,19^ skill development, and learning outcomes.^14-17^ Although qualitative considerations are important, there is a gap in the literature regarding economic impact analysis of DME in the Canadian setting. This paper therefore focusses on quantitative benefits.

There are methods identified in the literature to assess the economic impact of American DME programs, and broader studies assessing the economic impact of medical education in Canada^20^ or specific methodologies for medical programs in their entirety.^8^ For instance, the Northern Ontario School of Medicine (NOSM) study, measured the economic contribution of its education facility as well as the community members understanding of the benefits.^8^ The NOSM study was based on both economic data and qualitative data.^8^

The economic impacts of a DME program can accrue at different phases of DME development: conception, construction/creation, implementation, and evolution/consolidation.^8^ These impacts vary depending on the choice of one of three primary models of undergraduate DME in Canada, i.e., a clinical model, a longitudinal/distributed model, or a combined model.^2^ Economic impacts accruing to a community are contingent on the physical infrastructure developed in the region, educational supports secured there (i.e., preceptors and administration), the length of time students remain in the region, and the long-term impact of graduates who had trained in the region.^11^

An economic assessment method applicable to a variety of typologies and geographic contexts, e.g., smaller cities and rural communities, is needed to calculate impact of DME in these settings. The results of an economic assessment are useful for decision making by the community, university, and government stakeholders on the future of DME programs. We hypothesize that it is possible to modify an existing economic impact methodology for use in the DME context.

Approaches potentially used to assess economic impacts and contributions may be qualitative, statistical, mathematical, or a combination of the three. Qualitative approaches provide data and analysis beyond the dollar impact and reflect the context of the overall contribution of the DME program, such as the perceptions of the business and health communities. Although qualitative methods provide an important contribution to understanding benefits, and broader economic effects, a separate paper would be needed to capture the methods used to research these benefits. Statistical approaches are replicable and result in an economic impact in dollar values, but the complex nature of statistics and the skills required to run the programs make this approach more difficult to perform internally by small DME organizations. Moreover, we did not find any studies that had used a statistical approach. Mathematical approaches, in contrast, focus on the estimated dollar value of the economic impact based on spending, rather than perceptions, and are usually simpler to calculate. As a result, we focused this review on mathematical approaches. Different approaches were analysed, compared and evaluated for their potential to assess the economic impact of DME in Canada. Criteria included in the assessment of each method were: applicability to the Canadian context, data requirements and availability, the way the multipliers were calculated, information resulting from the calculations, replicability, scalability, and robustness.

Assessment methods using a mathematical approach are constructed using general economic principles or “economic base theory” or a combination of both.^8,21-23^ General economic principles offer a theoretical foundation for understanding economic impact. Based on a free market, resource allocation regulated by price, ability to pay, and complete information on the economy, general economic principles advocate that market forces will provide optimal allocation of healthcare resources.^23^ Economic base theory ensures the economic baseline for a site or region is measured in advance of an event, this base is then used to compare pre-DME with a post DME economy.^21^ However, measuring DME in terms of these economic principles is a challenge; general economic theory does not address market failures and society’s ethical attitudes toward health and provision of healthcare.^22^ Hence, we have explored other mathematical approaches in this study.

## Methods

We undertook a search of the peer-reviewed literature using a keyword search on Google Scholar (Canada + “distributed medical education” + economic impact). This identified only one published study in English relevant to our topic in Canada.^8^ We were also aware of a single study published in French.^21^ Given how few articles the researchers found on the topic, the search terms were then expanded to include: USA, international, Tripp Umbach, medical education. We also broadened the search to Google, to the capture grey literature, i.e., international studies and reports related to economic assessments of DME programs, medical schools, universities, and other studies that could be modified for the DME context. This second search did not result in more peer reviewed papers on the topic either in Canada or internationally. However, it did result in extensive consultant reports, i.e., 28 economic impact reports related to Canadian universities (21 individual university reports and seven more general or regional university studies), 22 reports on the economic impact of medical programs in the US most of which were prepared by Tripp-Umbach and two reports related to the cumulative economic impact of Canadian medical education.

Articles included in this study met the following inclusion/exclusion criteria:

Inclusion: Studies examining the economic impact of DME, a medical school, a group of medical schools or a university; studies including a model or method to assess economic impact of a university; and publications from peer reviewed and general literature if available as a full report.

Exclusion: Studies focussed on qualitative impacts, e.g., perceptions, attitudes and values; studies focused on one component of medical education, e.g., only students; and studies focussed on training medical professionals other than physicians; and reports that did not include a methodology

Although the authors recognize that socio-economic impacts are important, this paper focuses on quantitative economic impact assessment methods.

Brandon University Research Ethics Committee granted a research ethics exemption for this research paper as the information used was publically accessible and there was no reasonable requirement of privacy.

## Results

**Figure 1:**
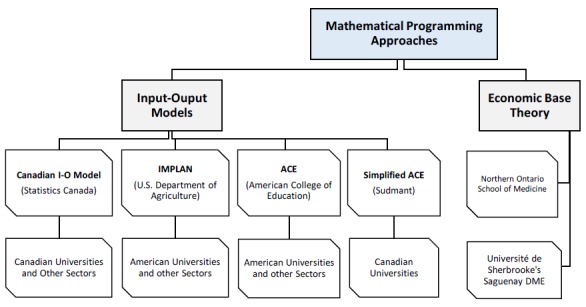
Economic impact assessment approaches with potential for DME in Canada

A review of the methodologies used in these reports found that the majority of the authors used an Input-Output model for their calculations. The two peer reviewed papers in contrast based their methodology on economic base theory. We found four different models in the I-O model category and one in the economic base theory category. All five models have the potential to be used to assess the economic impact of Canadian DME (refer to Figure 1).

### Input-Output models

The main model discussed in the literature derived from economic principles and theory is the I-O model.^24^ An I-O model determines the economic impact of an initial investment in the economy of a predefined area by tracking how the investment recirculates within the economic area depending on the interdependencies of a region’s industries.^23^ It utilizes region- and industry-specific multipliers (i.e., multipliers developed for either a specific province or a specific economic sector) to estimate how much of an initial investment (i.e., the direct effect) is re-spent by its suppliers across different industries (i.e., indirect effect) and by employees of the firm and its suppliers (i.e., induced effect). The total economic impact of the investment to the local economic area is the sum of the direct, indirect, and induced effect.^23^ An I-O model estimates employment impact: new jobs generated (in the region, in any industry) as a result of the initial investment. This model has been regularly used to assess university impacts and could be used to assess a portion of the university’s impact, i.e., the DME program.^25-27^

We provide a detailed description of four I-O models:

- *Canadian I-O Model*: Statistics Canada developed intraprovincial and national level Canadian I-O tables to estimate net economic activity generated from a specific enterprise or project.^28^ It then calculated multipliers from the I-O tables. These multipliers estimate the effect of “an exogenous change in final demand for the output of a given industry. The multipliers provide a measure of the interdependence between an industry and the rest of the economy.”^28^ These multipliers are subsequently used to determine the effect of an investment in one of 234 different industries, on gross output, gross domestic product (GDP) basic price, labour income, jobs, and indirect taxes.^28,29^ Studies using multipliers based on industry standards and averages are comparable within the same sector (e.g., health sectors in other locations) or with other industries. It is customary to assume impacts occur in a one-year time frame as they are based on annual data. These models require data inputs on university spending (both capital and operational), full-time students from outside the region, and visitor spending. It is also important to differentiate local and nonlocal spending. The Canadian I-O model has been used to assess the impact on the local economy of DME initiatives at the University of Manitoba, the University of Toronto-Mississauga, and the University of New Brunswick.^25-27^
- *IMPLAN Model:* The IMpact analysis for PLANning is an I-O model developed by the United States Department of Agriculture for community impact analyses.^30^ An I-O model offers a “snapshot” of the economy for a given period and region. Similar to the Canadian I-O Model, the IMPLAN model uses a spreadsheet system to identify all of the potential transactions of the industry under study and other industries. It is also used to measure shocks to a system; its basic premise is that supply matches demand. An increase in one of the variables will result in a “shock” to another part of the economic system. Output multipliers measure the cost of an additional dollar-unit of output to the final demand. Income multipliers measure the total change in income from a dollar-unit change in final demand for any given sector, and employment multipliers measure the total change in employment due to a one-unit change in the employed labour force of a particular sector source.^30,31^ The IMPLAN model has been used to evaluate the economic impact of rural health practices, for example, a general surgeon in rural United States,^32^ a rural residency program in Oklahoma,^33^ and a rural general practice.^34^ However, the application is limited to the U.S. context because the multipliers are American-based.
- *ACE Method:* The ACE Method is an alternative Canadian I-O model that was “developed by the American Council on Education (ACE) for the measurement of college and university economic impact.”^7^ It uses industry-generic, region-specific multipliers to estimate the economic impact of spending by a higher education institute (HEI), its faculty and staff, students, and visitors, on local businesses, governments, and individuals.^35^ The full ACE method includes such impacts as local banks’ expanded credit base as a result of HEI deposits and loss of real estate tax due to the tax-exempt statuses of HEIs. Because this is a data-intensive method, this approach is theoretically sound but practically difficult to apply. A common critique of the ACE method is that it may overestimate impact by not differentiating between local and non-local students, staff, and visitors. The economic impact is often measured as the result of “new” money, i.e., money that comes from outside of the region of interest.^35^ The ACE method, however, uses a counterfactual argument, that is, if an economic activity occurs in another location, this is a loss to the community. The ACE method documents an organization’s impact to an economy: an approximation of the economic activity (regarding jobs or dollars) in a specific region that accrues from the existence of a specific business, industry, or event.
- *Simplified ACE Method*: The authors of the Simplified ACE method use a single multiplier rather than many multipliers, used in the original ACE method. The Simplified ACE method has been used to evaluate the WWAMI Montana program.^7^ They determined a multiplier of 2.3 for the State of Montana; this was deemed appropriate for evaluating major research universities.^7^ The Simplified ACE method has also been used to estimate the combined economic impact of all 17 Canadian medical faculties and their affiliated teaching hospitals to the Canadian economy.^19^ This involved a review of the role of the healthcare and higher education sectors on the Canadian economy using Statistics Canada multipliers. They determined multipliers for business volume (2.5) and employment (1.8). Tripp Umbach calculated the direct effect of Canadian medical schools by including operating expenditures and spending by students and visitors. Their analysis concluded that Canadian Faculties of Medicine and teaching hospitals had a total economic impact of $66.1 billion and supported 295,000 jobs in the 2012/2013 Fiscal Year.^19^ The Simplified ACE method has also been used to evaluate the economic impact of the University of British Columbia (UBC) to the Province of British Columbia (BC).^36^ This study ignored the inter-industry dependence of an economy and used a single multiplier of 1.5. This multiplier measured the additional indirect and induced effects of all expenditures stemming from the existence of UBC (capital and operations expenditure, student spending, and visitor spending), regardless of the type of expenditure or who made it. By comparing the proportion of research conducted by UBC relative to the total value of research conducted in BC and multiplying this fraction by the annual change in BC’s economy, researchers calculated the total factor productivity, i.e., the economic effect of the innovation that results from the university’s research activity.^36^ This calculation yields the fraction of a change in economic growth attributable to UBC. To measure the alumni impact, Sudmant estimated the average difference in income of a university graduate compared to the average income of someone without a bachelor’s degree.^35^ This positive difference was attributed to the university. A counterfactual argument includes all full-time university students in the calculation of impact, i.e., if students did not attend a university locally, they would attend in another location. Hence, all full-time students are counted in the analysis regardless if they are local or from outside of the region.

### Economic base theory

Hogenbirk et al. assessed the economic contribution of the Northern Ontario School of Medicine (NOSM) in its entirety. The model they developed is built on economic base theory,^8^ which requires considerable longitudinal data. However, longitudinal data are not available for newer Canadian DME programs. The study ignored inter-industry economic activity and used a population-sensitive equation developed for Ontario to yield community-specific multipliers. These multipliers ranged from 1.02 in communities with populations of 2,500 to 1.79 in cities greater than 150,000.^8^ The direct effect included operating expenditures (salaries and benefits, travel, supplies and services, stipends paid to preceptors, student spending, and research expenditure) and excluded capital expenditures. These numbers were considerably lower than the 2.5 used by Tripp and Umbach^5^ in their Canada-wide study of medical programs but are similar to Sudmant’s multiplier of 1.5.^36^ The impact of the Saguenay DME program was estimated in terms of its impact on local income revenue and total employment following the NOSM method.^22^ The Université de Sherbrooke’s medical school developed DME programs on two sites, one in Moncton, New Brunswick, the other in Saguenay, Québec. Researchers calculated direct income revenue from the medical campus and estimated the impact of total (direct, indirect, and induced) income revenue in the region.^22^

Table 1, below, summarizes the findings of our literature review. We compared methods by: geographic context, data input and availability for input into the model, ease of calculations and information outputs, as well as more general qualities (i.e., scalability, replicability, and robustness). The parameters within each of these categories highlight specific similarities and differences between the methods and applicability to the Canadian DME context.

**Table 1:** Comparative table of the economic impact assessment methods

Models	Canadian I-O	IMPLAN	ACE	Simplified ACE	Economic Base Theory

**Application**					

Canada	X			X	X
United States		X	X	X	
Point-in-time	X	X			X
Long-term			X	X	

**Data**					

University spending	X	X	X	X	X
Research impact			X	X	
Alumni impact			X	X	
All students			X	X	X
Out of province students	X	X			
Simple data inputs			X	X	
Complex data inputs	X	X			X

**Multipliers**					

Use of single multiplier			X	X	
Industry based multipliers	X	X			
Fixed multipliers			X	X	
Updated multipliers	X	X			
Population Specific Multipliers					X

**Information Outputs**					

Single cumulative number			X	X	
Multiple by category	X	X	X	X	X
Comparison to other industry sectors	X	X			

**General Criteria**					

Scalability	X	X	X	X	X
Replicability	X	X	X	X	
Robustness	X	X	X	X	

## Discussion

Our review identified five potential models either previously used to assess economic impacts of DME programs or demonstrating the potential for use in the DME context. Table 1 shows how the models compare based on key criteria, which we will discuss in turn.

Specific criteria for evaluating economic models were not found in the literature, however, evaluation is typically tailored to a specific topic and standards of quality are delimited.^37,38^ Thus in a study assessing the potential of a model for the DME economic assessment there are standards that need to be met:

a) An appropriate methodology is needed to answer the research question: is the method or model applicable to the Canadian DME context?
b) Accessible and basic data are needed to prove a research question: are basic economic data available to run the method or model?
c) The calculations need to be clear and transparent: are the calculations to run each method or model transparent and replicable?
d) The information resulting from the calculations needs to be usable by decision makers: is the information produced by each model useful to decision makers and does it provide information on key economic impacts?
e) Robustness and scalability are requirements for a method or model that will be used for a wide variety of programs: Which methods are most likely to be used because they are robust and scalable to individual DME programs?

We evaluated each model to determine if it could be used in the Canadian DME context. Given the structure of the medical system, the difference in industries and subsequent development of the multipliers for the US context, the IMPLAN model and the ACE model are not a good fit for Canada, although some of the principles are applicable.

The I-O models and the economic base theory model calculate the economic impact for a single point in time, where both the ACE and the Simplified ACE calculate the dynamic impact over time by including the alumni and research impact. The I-O models can be updated every five years as the multipliers are updated. The Simplified ACE may be updated over time as impacts increase through an increase in students, teaching supports, alumni or research.

Data required to run each model were evaluated as well as the robustness of the model (i.e., the model should be able to adapt to some data not being available). Each method requires data on university spending and student numbers (e.g., students either from outside the region or all students). Given that many DME programs in Canada are relatively new, they may not have data available yet for all of the categories, and generally do not have high numbers of alumni or well-developed research programs. To use economic base theory to assess the economic impact of a DME, detailed information about the local population and economy is required. The Canadian I-O model and Simplified ACE model appear to be robust, i.e., the models can be run with minimal data, if data are incomplete a note is added to the calculation.

Multipliers are used in each of the models. The ACE and Simplified ACE use a single number for all categories. Both the IMPLAN and Canadian I-O models are linked to industry multipliers, thus are more complex to include in the calculations, as well they have to be updated regularly. The benefit is that these are based on industry standards and are replicable and ensure comparability of DME to other sectors. However, economic base theory depends on determining a specific population-based multiplier. This is a challenge especially if current data are not available; when faced with this circumstance studying NOSM, researchers estimated multipliers.^7^ The ACE and the IMPLAN models are based on the American context and their multipliers cannot be used to assess the Canadian DME economic impacts.

All of the models have been scaled to a smaller university and program-specific level. However, it is important, regardless of which model is used, to ensure that the limitations of the multipliers are stated. For instance, the I-O model multipliers are typically calculated at a provincial or national level and the ACE multipliers are standardized for each program under study regardless of the size of the region of impact.

Another key consideration is the replicability of the models, either for different DME programs or in different provinces. The Canadian I-O model and the Simplified ACE have been applied in different provinces. Given that the data inputs are standardized as are the calculations both models are replicable in subsequent years.

Thus there are two models, the Canadian I-O model and the Simplified ACE model, which meet the criteria outlined above, i.e., the models measure economic impact, data are available to input into the model, calculations are transparent and replicable, information resulting from calculations is useful for decision makers, and models are both scalable and robust to apply to a broad range of DME programs in Canada.

The Canadian I-O model and the Simplified ACE Model have been used to assess economic impacts of universities across Canada, in both smaller and larger city contexts. They are also easily scalable to a smaller subsection of the university, such as a DME program. The methodologies have a solid rationale underlying their application and a systematic method of analysis. The type of data required for each parameter to calculate the economic impact is explicit; this facilitates data gathering by the university to input into the model.

The inputs for the Canadian I-O model include university spending (i.e., operations, capital expenditure), full-time students from outside the region and their spending as well as visitor spending. The inputs for the Simplified ACE also include these parameters and all full-time students. Further to this, it also includes a calculation of the research impact and the alumni impact. These latter two inputs are included to capture the dynamic impacts of a university over time, rather than annual impact.

Both of the models use multipliers; however, the Canadian I-O model employs a different multiplier for each category of spending, based on industry averages. This requires more complex calculations and the use of Statistics Canada multiplier tables. These multiplier tables are updated every four years by Statistics Canada. The Simplified ACE, in contrast, uses a standard multiplier of 1.5 for all of the calculations; the multiplier remains constant over time, and therefore, is much easier to apply and needs updating only when there is a major change in one of the inputs.

To apply the Canadian I-O model, researchers need to classify spending using one of the categories linked to a specific multiplier on the Statistics Canada tables and determine if spending is local or outside the region. The Simplified ACE, in contrast, requires tallying spending by category and applying a single multiplier to all data. There is a standard method for calculating the research impact.^39^ The alumni impact is calculated by multiplying the number of alumni (both undergraduate and graduate) by the average of the annual wage increase attributed to completing a university degree.

The Canadian I-O model assesses the economic impact for total gross output, gross domestic product basic price, labour income, jobs created and indirect taxes. These results are not cumulative. Each stakeholder incorporates the output needed for the decision-making process.^3^

The simplified ACE method calculates outputs for each category of spending, e.g., university, student and visitor, and alumni and research impact. Alternatively, categories are summed together thus resulting in a single number. Depending on the type of decision, either information output is useful for decision-making.

Although decision-makers may be interested in the full impact of the DME, the multiple stages of development for a DME program challenge the analyst to provide a complete economic impact assessment, at least initially. For example, student numbers tend to increase as a program reaches capacity. Each year of a program will add a new cohort of students. If the cohort is 40 students, year one will comprise 40 students, and year two will comprise 40 new, students, thus in a three-year program, a total of 120 students will be enrolled each year. Documenting differences for each year will ensure measurement of per student impact and the overall program impact at maturity. Economic impacts may not be fully appreciated for new DME programs or if the physician shortage is addressed early by physicians relocating to the region to teach in the DME program. The economic impact (output) will rely on the magnitude of input and subsequent output (e.g., graduated physicians remaining in the region). As physicians are among the top Canadian income earners, any increase in their local or regional numbers will affect the magnitude of the economic contribution. Studying DME impacts at each stage of development will document the long-term impact from the conception stage to full DME implementation.

Although this literature review fills a gap by identify potential methods to quantitatively assess the impact of DME, this approach only addresses part of our understanding of the overall impact of DME, the qualitative impacts such as the perceptions of the community and business leaders as well as the general public may have an equal if not greater impact on the expansion or future of a DME program in a community. These qualitative data are especially important to decision makers as there are limited dollars available for medical education. Given that the role of DME programs is to also address the maldistribution of physicians, decision making needs to include a broader perspective.

The models in this review calculate the point-in-time and dynamic economic impact, however, as DME evolves through the phases of conception to maturity, economic impacts change. It is important for future users of these models to recognize their limitations. For example, for new DME programs the Canadian I-O model appears to provide the best point-in-time data, however, as research capacity expands and alumni remain in underserved areas as practicing physicians, the decision makers may consider a Simplified ACE that includes these two parameters. Thus, periodic economic assessments to capture this evolution of DME should be conducted to help develop an understanding of economic impacts over time. Further to this, data required to input into the model may be considered proprietary and/or not be specifically allocated to the DME, thus it may be a challenge to extract data specific to a DME program.

Given the broad range of DME programs, from small campuses where the majority of training takes place to fully distributed, where training takes place in many small communities, it may be difficult to gather all of the data required to calculate the economic impact of a DME. This complexity may be compounded by changing student numbers or students training over multiple fiscal years.

To enhance our understanding of the impact of DME, these models should be tested using a variety DME programs and at the different stages of maturity. This will help researchers understand their strengths and limitations, i.e., data that are easily available versus unattainable. Further to this, models need to be developed to include a component that measures the economic impact of graduates post DME. Practicing graduates may have a large economic impact in small communities, and remaining in communities may lead to other medical services. Another area of research could include measuring the economic benefits accrued to the community by the augmentation of local services due to physicians remaining in a region. Patients remaining in the local area to receive medical services, may pass on personal savings on transportation and accommodation, to other sectors of the local economy.

### Conclusion

Our review of methods to assess the economic impacts of Canadian DME programs identified the Canadian I-O model and the Simplified ACE method as the best candidates.^28,40^ These two models fit criteria needed for the DME program, e.g., applicable to the Canadian DME context, data inputs, applicable multipliers, useful information outputs and general criteria of replicability, robustness and scalability to the DME context.

Although the Canadian I-O model appears more precise because of the industry-based multipliers, there is a degree of estimation in determining the allocation of spending in different categories in the region and number of visitors. The Canadian I-O model also has an advantage as the multipliers used in this model are also used in other industry sectors. This means that studies on DME are more easily comparable to other economic sectors. One of the key benefits of the outputs of a Canadian I-O model is its use for decision makers: the results provide information on Gross Output, GDP Basic Price, Labour Income, Total Jobs and Total Indirect Taxes.

The Simplified ACE method is dynamic and helps to understand the long-term impacts of a DME program on a local community from both the initial training (e.g., funding of preceptors, student, and visitor spending), to the benefit of students remaining in rural areas, thus addressing the maldistribution of medical doctors. Furthermore, as DME programs evolve and develop research programs, this model can capture the economic impact of the research conducted by faculty and students. The disadvantage is the use of a single multiplier regardless of the category of spending or region size. However, the simplicity assists in the calculation of an overall value.

Thus, the economic impacts can be captured through analysis at each phase of DME development and should be conducted at regular intervals. Documenting the increased attraction/retention of physicians (alumni and others) predicted by the DME philosophy helps to assess the full impact of DME. DME programs may take 5-10 years to have a significant impact from the initial enrolment, which means that impact may increase considerably over time. The Simplified ACE method is easily updated at low cost, providing a trend analysis over time and a more detailed picture of the overall impact. The authors strongly believe regular analysis over the long-term is necessary of an economic impact study. The relationship between cause and consequences of impact may be lost if only a single study is conducted, i.e., the DME program becomes a part of the normal situation, is taken for granted and thus forgotten, or data are no longer available.

The next steps of this research are to build a body of case studies at various stages of DME development and to examine more fully the other qualitative methods to measure benefits. With the application of these economic assessment methods to different DME programs, the benefits and challenges of each method will become apparent, which should assist future analysts to collate and calculate information on the economic impact of DME programs in Canada. An assessment of qualitative methods to measure social impacts should also provide a greater understanding of benefits beyond the fiscal that can act as catalysts to improve medical education and services in communities.
